# Exogenous Cytokinin 4PU-30 Modulates the Response of Wheat and Einkorn Seedlings to Ultraviolet B Radiation

**DOI:** 10.3390/plants13101401

**Published:** 2024-05-17

**Authors:** Elisaveta Kirova, Irina Moskova, Vasilissa Manova, Yana Koycheva, Zoia Tsekova, Denitsa Borisova, Hristo Nikolov, Ventzeslav Dimitrov, Iskren Sergiev, Konstantina Kocheva

**Affiliations:** 1 Institute of Plant Physiology and Genetics, Bulgarian Academy of Sciences, Acad. G. Bonchev Str., bl. 21, 1113 Sofia, Bulgaria; elisab@abv.bg (E.K.); irinamoskova@yahoo.com (I.M.); yana.koicheva@gmail.com (Y.K.); kmeti@abv.bg (Z.T.); iskren@bio21.bas.bg (I.S.); 2 Space Research and Technology Institute, Bulgarian Academy of Sciences, Acad. G. Bonchev Str., bl. 1, 1113 Sofia, Bulgaria; dborisova@stil.bas.bg (D.B.); hristo@stil.bas.bg (H.N.); vdimitro@stil.bas.bg (V.D.)

**Keywords:** antioxidant gene expression, flavonoids, oxidative stress, phenolic compounds, phenylurea-cytokinin, proline, remote sensing, ROS, spectral reflectance

## Abstract

Abiotic stress is responsible for a significant reduction in crop plant productivity worldwide. Ultraviolet (UV) radiation is a natural component of sunlight and a permanent environmental stimulus. This study investigated the distinct responses of young wheat and einkorn plants to excessive UV-B radiation (180 min at λ_max_ 312 nm) following foliar pretreatment with 1 µM synthetic cytokinin 4PU-30. Results demonstrated that UV radiation significantly amplified hydrogen peroxide levels in both wheat and einkorn, with einkorn exhibiting a more pronounced increase compared to wheat. This elevation indicated the induction of oxidative stress by UV radiation in the two genotypes. Intensified antioxidant enzyme activities and the increased accumulation of typical stress markers and non-enzyme protectants were evidenced. Transcriptional activity of genes encoding the key antioxidant enzymes POX, GST, CAT, and SOD was also investigated to shed some light on their genetic regulation in both wheat and einkorn seedlings. Our results suggested a role for *POX1* and *POX7* genes in the UV-B tolerance of the two wheat species as well as a cytokinin-stimulated UV-B stress response in einkorn involving the upregulation of the tau subfamily gene *GSTU6*. Based on all our findings, it could be concluded that 4PU-30 had the potential of alleviating oxidative stress by attenuating the symptoms of superfluous UV-B illumination in the two examined plant species.

## 1. Introduction

Wheat (*Triticum aestivum*, L.) stands among the first plant species domesticated by mankind and has become a crop of key agronomical importance nowadays, supplying the primary food sources for humans worldwide [1]. In recent years, young cereal seedlings have been employed in the health food industry as an innovative and fashionable alternative to tasty ready-to-eat vegetables, rich in various bioactive compounds [2]. The growing interest in healthy nutrition diets and improved quality of life determines the pursuit of specialized food products based on alternative cereal species like einkorn and emmer. Einkorn (*Triticum monococcum*, L.) is an ancient wheat species which possesses superior nutritional value and better technological characteristics of the grain compared to extensively grown modern wheat varieties [1,3,4]. Despite their lower seed productivity, ancient wheats have survived in adverse environmental conditions for many years and are presumably more resistant to stress factors compared to cultivated wheat, offering possibilities for their implementation in organic farming [5]. 

Sudden changes in environmental factors have a negative impact on plant growth and development. Abiotic stress may arise in response to soil salinity, water deprivation, high temperatures, or a combination of multiple factors. Although a natural component of sunlight and a portion of the non-ionizing region of the electromagnetic spectrum (comprising about 9% of the emitted solar radiation), ultraviolet (UV) radiation is a strong environmental signal and a powerful abiotic stress factor [6]. While mostly attenuated by the stratospheric ozone layer, part of UV-B radiation (280–315 nm) penetrates deeper, reaching the Earth’s surface and is more dangerous due to its high energy and ability to interact with organic molecules. Exposure to excessive UV-B could be highly damaging for the plant at many organizational levels. At the genomic level, UV-B light generates DNA lesions, predominantly pyrimidine dimers, that can directly block DNA replication and RNA synthesis [7,8]. Additionally, UV-B induces the formation of reactive oxygen species (ROS), which are highly active and cause the oxidation of key cellular components such as proteins and lipids, eventually leading to oxidative stress. At a higher organizational level, UV-B impairs the structure and functioning of the photosynthetic apparatus, causing leaf wilting, yellowing, and even death of the whole plant organism [9,10]. 

Plants have evolved a variety of mechanisms to limit UV-B-induced lesions and promote acclimation to excessive radiation [6,11]. Defense resources for coping with the negative consequences of oxidative stress are aimed to detoxify ROS, reduce cellular damage, and facilitate DNA repair. These involve the biosynthesis of both enzymatic and non-enzymatic protective molecules. Although the accumulation of low molecular solutes like proline, sugars, and polyols is a common stress response, it could hardly minimize oxidative injury, so these substances are fairly regarded as stress markers [12]. Another endogenous cellular constituent, malondialdehyde (MDA), is frequently used as a stress reporter molecule representing a product of lipid peroxidation [13,14]. Non-enzymatic protection also comes from the increasing levels of biological compounds such as ascorbic acid, phenolics, flavonoids, glutathione, and proteins that carry redox-active S-groups and act as antioxidants. On the other hand, enzymes involved in antioxidant protection limit the consequences of oxidative stress by their ability to scavenge free radicals and toxic amounts of ROS either through direct decomposition and balancing cellular redox dynamics or preventing their formation by regulating gene expression and proteolysis [13].

Cytokinins (CKs) are phytohormones involved in various aspects of plant growth and development including cell division and differentiation and shoot and root morphogenesis, but they have also been shown to mediate plant stress responses [15,16,17,18,19,20]. The synthetic phenylurea type cytokinin 4PU-30 (N1-(2-chloro-4-pyridyl)-N2-phenylurea) has been reported to reduce the negative effects of drought [21,22] and salinity [23,24], alleviate the action of the herbicide glyphosate [25,26], and provide protection against herbicides and biotic stress [25,27]. The application of growth regulators capable of activating the plant defense system might be regarded as an alternative approach to developing stress tolerance in important crop species [28,29]. 

Changes in gene expression related to antioxidant enzyme activities are regarded as a hallmark of plant oxidative stress tolerance. Modulated expression patterns of the genes encoding antioxidant enzymes such as catalase (CAT), peroxidase (POX), superoxide dismutase (SOD), and glutathione S-transferase (GST) in response to enhanced ROS production have been reported in several plant species, including wheat, after exposure to a variety of stressors [30,31,32,33,34]. *SOD* genes have been evidenced as highly responsive to abiotic stress, thus enhancing plant resistance. Their individual expression patterns, however, have revealed stress-specific differences and variations among species [35,36]. UV-B exposure has been shown to trigger the dynamic transcriptional activity of *SOD* genes with significant upregulation observed only for certain mRNAs [37], suggesting that the regulatory role at the gene expression level is crucial for establishing stress tolerance [38]. Increased transcript levels were found in einkorn plants subjected to heavy metal toxicity, which was consistent with the stress-responsive cis-regulatory elements identified in the promoter region of *TmMnSOD* [39]. Accumulated experimental data indicated that at the transcriptional level, most of the antioxidant genes exhibited high plasticity and complex regulatory features, allowing the plants to properly respond and precisely activate their antioxidant defense system [40,41,42].

The aim of the present work was to compare the physiological responses of young wheat and einkorn plants to excessive UV-B radiation in laboratory experiments and to explore the capacity of exogenously applied synthetic phenylurea type cytokinin 4PU-30 for alleviating oxidative stress damage. Furthermore, the transcriptional activity of genes encoding certain antioxidant enzymes was measured to explore the genetic regulation of the antioxidant defense system. An additional focus of this paper was to use the possibilities of narrow-wavelength spectrometric measurements and to obtain spectral data in the monitoring of wheat and einkorn plants under UV-B stress. For this purpose, a remote sensing (RS) approach was applied which was previously tested in different experiments [43,44,45]. Here, spectral reflectance data acquired by a multichannel spectrometric system were used for establishing a relation with unfavorable stress caused by UV-B radiation. 

## 2. Results

### 2.1. Effect of Cytokinin Priming and Subsequent UV-B Treatment on Growth Parameters in Wheat and Einkorn Seedlings

**Figure 1:**
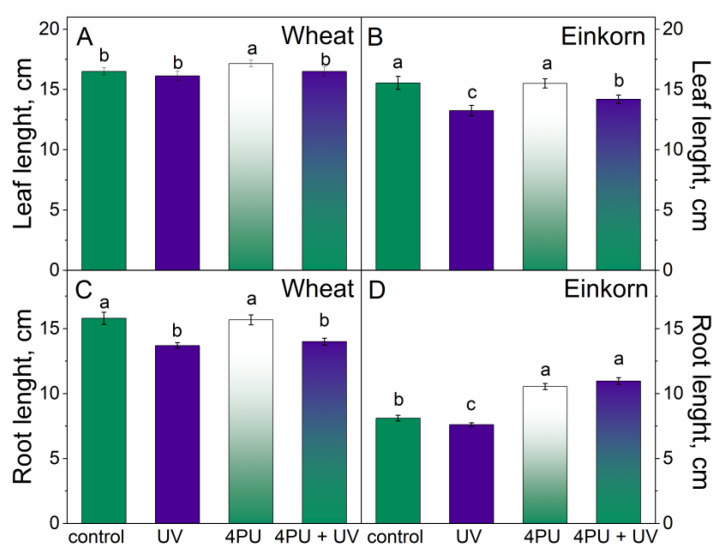
Leaf (**A**,**B**) and root length (**C**,**D**) of wheat and einkorn seedlings treated with cytokinin 4PU-30 and subjected to subsequent UV-B radiation. Results are given as means ± SD (n = 20). Different letters represent statistical significance at *p* < 0.05.

UV-B treatment caused a decrease in leaf length only in einkorn but not in wheat (Figure 1A,B) and reduced root length most notably in wheat plants (Figure 1C,D). 4PU-30 pretreatment led to increased leaf length in wheat and increased root length in einkorn plants (Figure 1A,D). Wheat plants treated with UV-B after cytokinin imposition showed slightly reduced root length compared to controls (Figure 1C), while in einkorn, the combination of the two treatments led to increased root length (Figure 1D). 

Pigment content in the leaves decreased after UV-B treatment in all variants (Table 1). Chlorophyll a and b and carotenoids were more significantly reduced in wheat compared to einkorn leaves. Generally, 4PU-30 caused an increase in chlorophylls and carotenoid amounts. Additionally, the cytokinin had positive effect on all pigments’ quantities after successive UV-B treatment compared to variants solely illuminated with UV-B radiation.

### 2.2. Effect of 4PU-30 and UV-B Treatment on Reflectance Spectra of Wheat and Einkorn Seedlings

**Figure 2:**
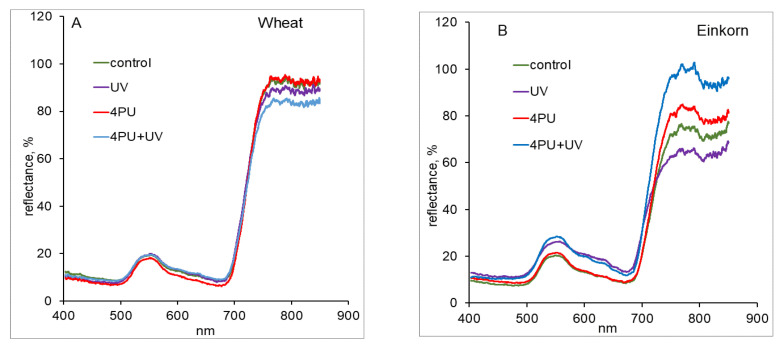
Averaged reflectance spectra of wheat (**A**) and einkorn (**B**) plants treated with excessive UV-B radiation and sprayed with synthetic cytokinin 4PU-30 separately and in combination.

To study the dynamics of wheat and einkorn control and treated plants at a leaf level, their reflectance spectra were obtained by remote sensing measurements. Intact, photosynthetically active plants usually exhibit a typical pattern of their spectral characteristics. Normally, a small peak in the green region of the visible spectrum (520–580 nm) is found that corresponds to the maximal chlorophyll reflectivity. After that, a decrease in the red range (640–680 nm) is seen, which is related to high chlorophyll absorption. Next, a well-manifested steep increase can be observed in the so-called “red edge”, a range of the electromagnetic spectrum (EMS) between 690 and 720 nm. In the near/shortwave infrared (NIR/SWIR) parts of EMS (720–2500 nm), the reflectance remains high up to 1200 nm and subsequently starts to decrease. Both “red edge” and NIR/SWIR are considered as the most informative regions of EMS for studying plants, which was clearly expressed in the wheat and einkorn averaged reflectance spectra (Figure 2A,B). It was seen that in wheat, 4PU-30 did not affect the obtained reflectance spectra, while UV-B treatment and its combination with preliminary 4PU-30 application caused a decrease in the reflectance response in the NIR region (Figure 2A). In einkorn, UV-B and its combination with 4PU-30 induced a significant increase in reflectance in the green spectral range (520–580 nm) but not in the “red edge” and NIR ranges (Figure 2B). In the NIR part of the EMS, exposure to UV-B led to a decrease in the reflectance, while 4PU-30 treatment and its combination with UV-B resulted in a visible increase in the registered reflectance (Figure 2B).

### 2.3. Effect of 4PU-30 and UV-B Treatment on Stress Markers Content in the Leaves of Wheat and Einkorn Seedlings

**Figure 3:**
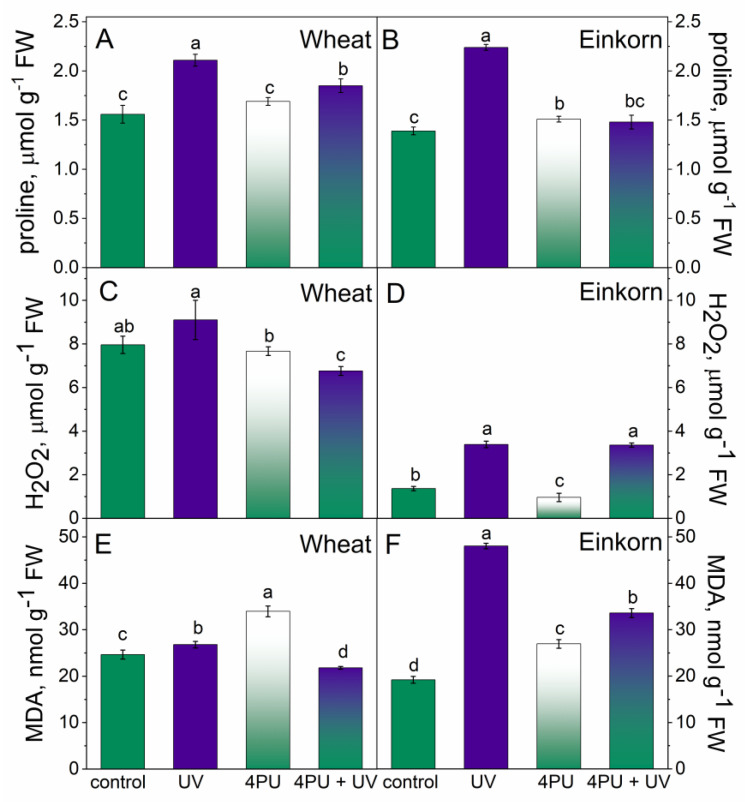
Oxidative stress markers in the leaves of wheat and einkorn plants treated with excessive UV-B radiation and sprayed with synthetic cytokinin 4PU-30. Amounts of free proline (**A**,**B**); hydrogen peroxide, H_2_O_2_ (**C**,**D**); and malondialdehyde, MDA (**E**,**F**), given as means ± SD (n = 6), different letters represent statistical significance at *p* < 0.05.

Analyses of oxidative damage and antioxidant status were performed on the first fully developed leaf of control, UV-B-treated, and cytokinin-primed plants. Cytokinin priming had a slightly supplementary effect in einkorn and no significant effect in wheat with respect to proline content. UV-B treatment caused an increase in free proline levels in both plant genotypes, but it was greater in einkorn when compared to the respective controls (Figure 3A,B). Following UV-B illumination, proline content was increased up to 35% in wheat and 61% in einkorn compared to the corresponding untreated plants. Proline values in einkorn seedlings subjected to a combination of 4PU-30 and consecutive UV-B were lower compared to UV-B-treated variants and similar to controls and cytokinin-sprayed plants. In wheat, the combined treatment with cytokinin and UV-B stress led to a slight increase in proline levels. 

Cytokinin spraying did not significantly affect hydrogen peroxide levels in wheat and slightly reduced them in einkorn (Figure 3C,D). UV-B treatment led to an increase in hydrogen peroxide values, which were 30% higher than controls in wheat and nearly 1.5-fold above einkorn controls (Figure 3C,D). The combination of cytokinin pretreatment followed by UV-B illumination decreased H_2_O_2_ in wheat but increased it in einkorn with respect to controls. 

The amounts of MDA usually indicate the degree of membrane lipid peroxidation resulting from oxidative damage. MDA content only slightly increased in wheat after UV-B treatment but exhibited a more than 2-fold growth in einkorn compared to the respective controls (Figure 3E,F). Cytokinin spraying, prior to the UV-B treatment, reduced the amounts of this oxidation product in wheat compared to the control and post-UV stress values; however, in respective einkorn variants, MDA remained higher than controls. 

### 2.4. Non-Enzymatic Antioxidants (Free Thiols, Flavonoids, and Total Phenolic Compounds)

**Figure 4:**
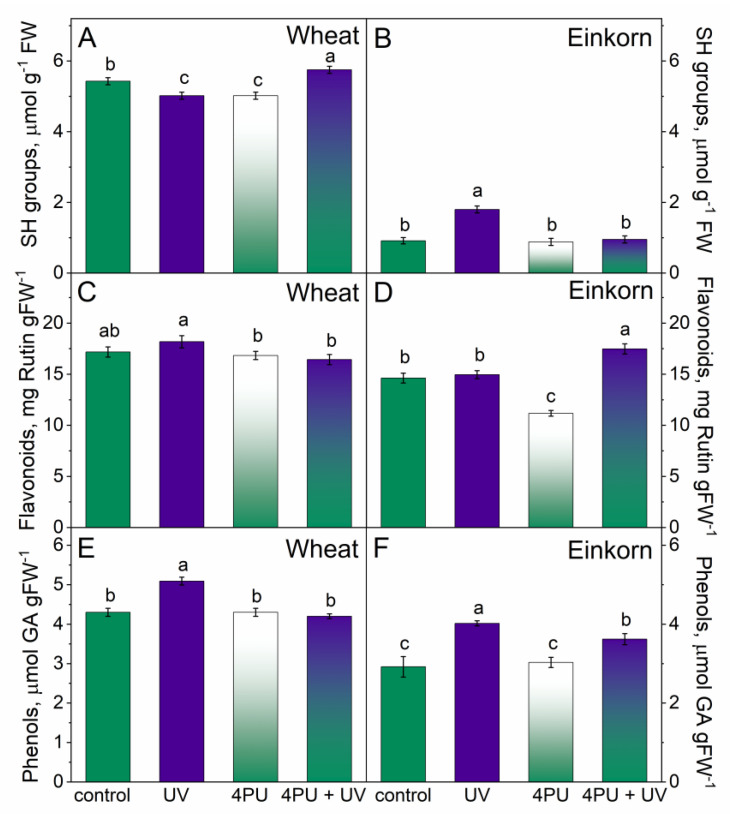
Non-enzyme antioxidants in the leaves of young wheat and einkorn plants illuminated with UV-B, sprayed with synthetic cytokinin 4PU-30 and a combination of the two treatments. Amounts of free thiol compounds, SH groups (**A**,**B**); flavonoids (**C**,**D**); and total phenolic compounds (**E**,**F**) in wheat and einkorn, respectively, given as means ± SD (n = 6). Different letters represent statistical significance at *p* < 0.05.

The amount of free thiol group-containing compounds, flavonoids, and total phenolics was measured in the first fully developed leaves of young wheat and einkorn plants. In wheat, the levels of SH groups remained relatively unaffected by the applied treatments; though a nearly 2-fold increase in free thiols compared to the control was detected in einkorn plants after UV-B irradiation. However, they returned to the controls after the combined action of 4PU-30 and illumination (Figure 4A,B). 

Flavonoid content was only slightly affected by UV-B treatment in wheat. In einkorn, cytokinin spraying evoked a 23% decrease in flavonoids and the consecutive treatment of cytokinin and UV-B led to a 20% increase in these cellular compounds (Figure 4C,D). 

Higher levels of phenolic compounds were measured in both genotypes following UV-B illumination, yet a more pronounced rise was found in einkorn, 37.7%, than in wheat, 18%, compared to the respective controls (Figure 4E,F). 4PU-30 application and subsequent UV-B illumination reverted the amounts of these substances to the control values in wheat and left them slightly higher than the controls in einkorn.

### 2.5. Free Radical Scavenging and Antioxidant Activity

**Figure 5:**
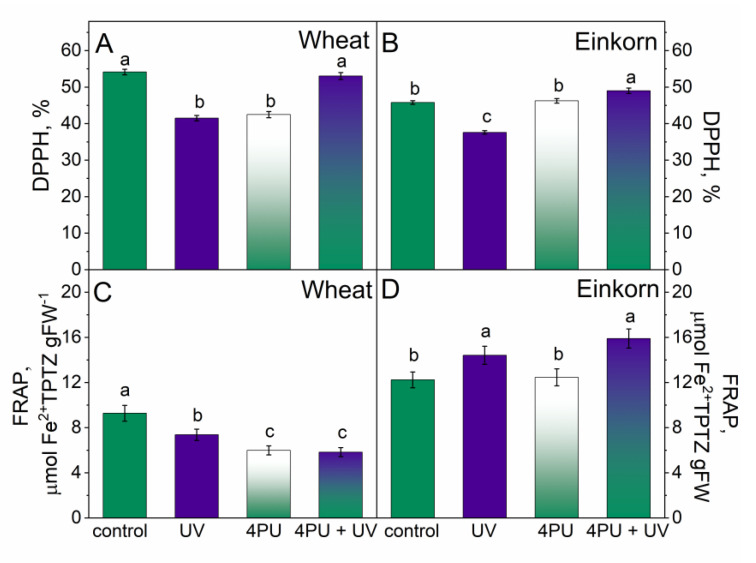
Free radical scavenging activity measured by the level of DPPH reduction in % (**A**,**B**) and antiradical activity expressed as ferric reducing antioxidant power (FRAP) (**C**,**D**) in the leaves of young wheat and einkorn plants illuminated with UV-B, sprayed with synthetic cytokinin 4PU-30, and a combination of the two treatments. Data are given as means ± SD (n = 6). Different letters represent statistical significance at *p* < 0.05.

In both genotypes, free radical scavenging activity estimated from DPPH• neutralization was reduced in response to excessive UV-B radiation, though the reaction was more pronounced in wheat (Figure 5A,B). Leaf spraying with 4PU-30 prior to UV-B treatment had a positive effect in both genotypes and resulted in preserved activity in wheat and slightly increased in einkorn compared to the respective controls. 

Antioxidant activity, expressed as FRAP, was reduced in wheat and enhanced in einkorn after UV-B radiation. Cytokinin spraying also reduced this parameter in wheat and had no significant impact in einkorn. The combination of treatments lessened the antioxidant activity in wheat and improved it in einkorn as compared to the respective controls (Figure 5C,D). 

### 2.6. Activity of ROS-Scavenging Enzymes (SOD, CAT, POX, and GST) 

**Figure 6:**
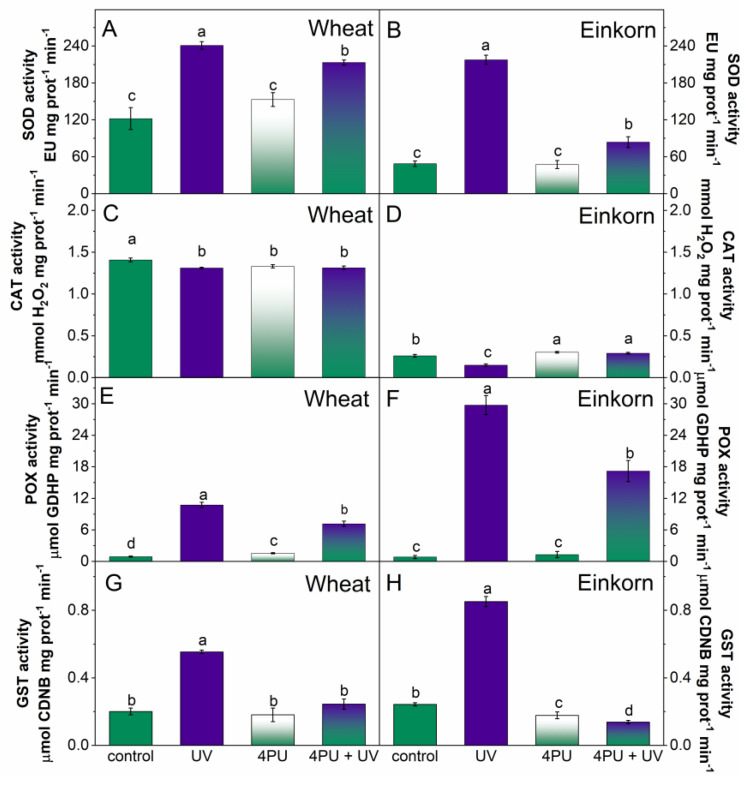
Activity of antioxidant enzymes in the leaves of young wheat and einkorn plants illuminated with UV-B, sprayed with synthetic cytokinin 4PU-30 and a combination of the two treatments. Superoxide dismutase, SOD (**A**,**B**); Catalase, CAT (**C**,**D**); Guaiacol peroxidase, POX (**E**,**F**); Glutathione S-transferase, GST (**G**,**H**). Data are given as means ± SD (n = 6). Different letters represent statistical significance at *p* < 0.05.

UV-B treatment caused an almost 2-fold increase in the activity of SOD in wheat and 4.5-fold increase in einkorn. Cytokinin pretreatment significantly lowered the effect of UV stress in einkorn and had a visible influence on SOD activity in wheat as well (Figure 6A,B). 

CAT activity in the leaves of wheat plants was only slightly reduced by UV-B treatment, cytokinin spraying, and their mutual application. In einkorn, CAT activity decreased to 57% of controls due to UV-B illumination; however, cytokinin pretreatment displayed a positive effect by almost reverting this enzyme activity to control levels (Figure 6C,D).

POX activity was greatly increased by UV-B treatment and was 12 times higher than controls in wheat and 35 times higher than einkorn controls. 4PU-30 had a certain recovering effect in both wheat and einkorn but could not bring POX activities to their respective control values in either of the genotypes (Figure 6E,F). 

GST exhibited elevated activity after UV-B treatment with a nearly 3-fold rise above control levels in wheat and a 3.5-fold increase in einkorn. 4PU-30 exerted an obvious recovery effect on GST enzyme activity when applied before the UV-B treatment, and reverted GST to its control activities in both genotypes (Figure 6G,H).

### 2.7. Gene Expression Analysis

**Figure 7:**
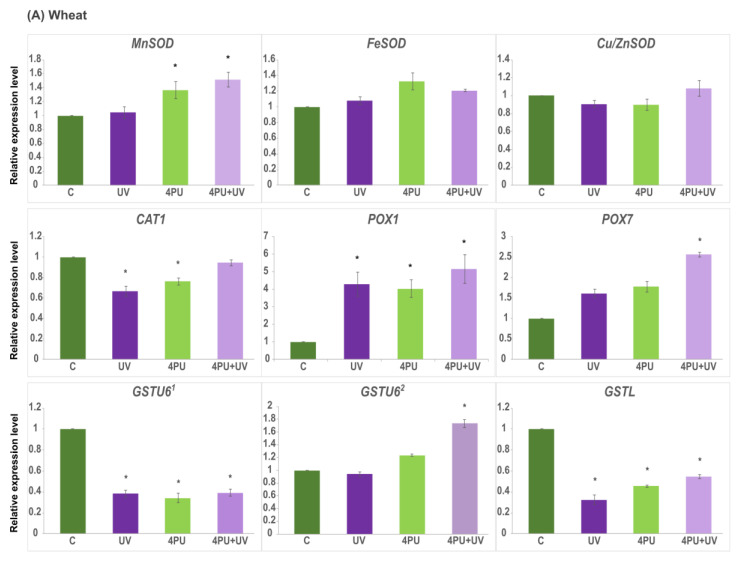

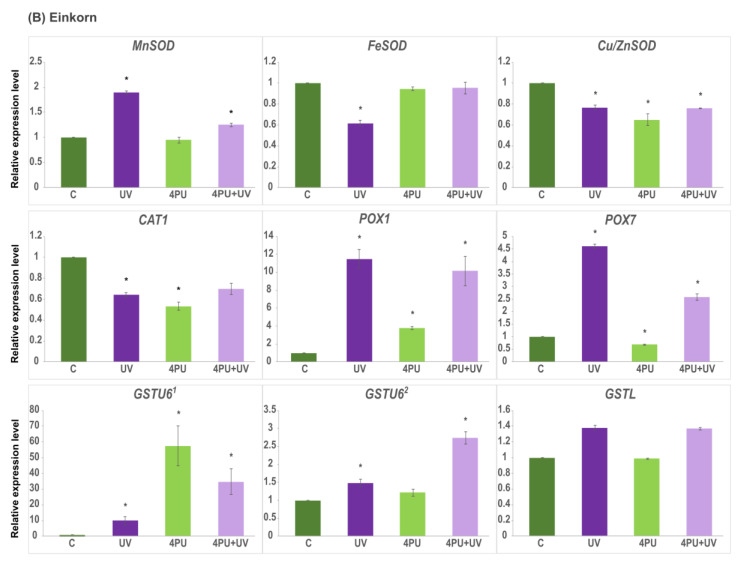
Relative expression levels of antioxidant genes *MnSOD*, *FeSOD*, *Cu/ZnSOD*, *CAT1*, *POX1*, *POX7*, *GSTU6^1^*, *GSTU6^2^*, and *GSTL* analyzed in wheat (**A**) and einkorn (**B**). Control (C), UV-B exposure (UV), application of 1 µM 4PU-30 (4PU), and combined treatment with UV-B and 1 µM 4PU-30 (4PU + UV). Results are presented as mean ± SE, n = 3. Asterisks (*) indicate significant difference compared to controls (*p* ≤ 0.05).

The results showing the effect of UV-B treatment and 4PU-30 application on the expression levels of genes encoding antioxidant enzymes are presented in Figure 7. The transcript abundance was assessed 72 h after UV-B irradiation in wheat and einkorn seedlings, pretreated or not with the cytokinin regulator 4PU-30 at a concentration of 1 μM. The relative expression levels of three genes belonging to the three groups of *SODs* (*MnSOD*, *FeSOD*, and *Cu/ZnSOD*), one *CAT* gene (*CAT1*), two *POX* genes (*POX1* and *POX7*), and three *GST* genes—*GSTL* and two different *GSTU6* genes designated as *GSTU6^1^* (localized in wheat chromosome 6A) and *GSTU6^2^* (residing in 7A and 7D, see Table 2)—were analyzed. The *MnSOD* gene was significantly upregulated in einkorn plants subjected to UV-B irradiation (Figure 7B), as well as in the 4PU-30-pretreated wheat and einkorn plants exposed to UV-B (Figure 7A,B). On the contrary, UV-B irradiation downregulated the expression of *FeSOD* and *Cu/ZnSOD* in einkorn but had no effect on wheat seedlings (Figure 7B). UV-B radiation and 4PU-30 application decreased the expression of *CAT1* gene in both species, although this effect was more evident in einkorn (Figure 7A,B). The relative transcriptional activity of the *POX1* gene was increased in both wheat and einkorn in all studied groups compared to the controls (Figure 7A,B). The increase in *POX1* mRNA levels was more pronounced in einkorn than in wheat samples, especially after UV-B and combined treatment (Figure 7B). A similar pattern of overexpression was observed for the *POX7* gene after UV-B irradiation in einkorn, though the plant regulator alone caused only slight downregulation (Figure 7B). Both in einkorn and wheat, *POX7* expression increased after successive 4PU-30 and UV-B exposure (Figure 7A,B). The *GST* genes showed differential expression depending on the sample and the gene analyzed. In einkorn, *GSTU6^1^* was upregulated in all studied groups compared to the control, with the most dramatic increase after cytokinin application (Figure 7B). On the contrary, in wheat plants, the expression of *GSTU6^1^* was downregulated in all samples (Figure 7A). The expression pattern of *GSTU6^2^* was similar in the two plants studied. A slight increase was observed after UV-B irradiation in einkorn (Figure 7B), and in both einkorn and wheat, a marked increase in *GSTU6^2^* transcripts was detected after combined cytokinin and UV-B treatment (Figure 7A,B). The expression of the *GSTL* gene differed between the two species with no substantial changes in the einkorn samples (Figure 7B), whereas in wheat plants, *GSTL* was significantly downregulated in all variants compared to the control (Figure 7A).

## 3. Discussion

Unfavorable environmental conditions significantly reduce crop productivity, thus challenging food security on a global scale. While UV-B radiation is a natural abiotic factor with a key role in plant life cycle, at higher doses, it can potentially damage and impair plant performance [46,47]. CKs are plant hormones which moderate growth and development under normal conditions; however, they are also acknowledged as active participants in plant responses to stress such as heat, drought, cold, and excessive salinity [20,28,48,49]. Studies have revealed that CKs increased leaf chlorophyll content, chloroplast stability and net photosynthetic rate, thereby alleviating stress-induced damages [49,50]. The application of growth regulators for improving abiotic stress response is a promising strategy in modern agriculture and efforts are aimed at characterizing the molecular mechanisms underlying their mode of action [23,28,51].

In the present study, we examined the capacity of exogenously applied synthetic cytokinin 4PU-30 for minimizing the consequences of oxidative stress in wheat and einkorn seedlings induced by excessive UV-B radiation. Our results demonstrated that cytokinin priming stimulated root growth in einkorn and ameliorated leaf pigment content in UV-B-treated plants of both genotypes, diminishing the inhibitory effect of radiation on these parameters. Although cytokinin pretreatment could not fully recuperate the reduction in leaf length caused by UV-B, nonetheless it had some supplementary effects on growth. These findings are consistent with the previously described effect of CKs promoting root elongation [52,53], as well as recent studies revealing developmental and morphological adjustments in response to UV-B stress [54]. Additionally, the exogenous application of the growth regulator melatonin was reported to significantly alleviate the inhibitory effect of UV-B on root growth [55]. 

The averaged reflectance spectra, obtained in our study by remote sensing measurements, demonstrated a characteristic peak around 550 nm in the green range of the EMS, which was higher for einkorn plants treated with UV-B and its combination with 4PU-30, and corresponded to the higher chlorophyll content in einkorn compared to the same wheat variants. Moreover, einkorn was affected to a higher degree by UV-B compared to wheat, though when treated with 4PU-30, einkorn plants recovered better than wheat plants. UV-B caused a significant decrease in chlorophyll and carotenoid content while 4PU-30 treatment resulted in higher pigment values in both studied plants. Our results are in agreement with previous findings showing that the reflectance measured in the visible region is generally affected by the concentration of leaf photosynthetic pigments (chlorophylls, carotenoids, and anthocyanins), while in the NIR region, it is mostly due to light scattering inside the leaf and therefore related to anatomical traits like mesophyll thickness and stomata structure [56,57,58]. Therefore, the differences in the reflectance spectra between wheat and einkorn observed in the NIR region probably represent a genotypic response. Control wheat plants exhibited higher values in the NIR region of the EMS compared to einkorn, while in the visible part of the EMS, their spectral characteristics were similar. This effect could be attributed to the denser structure of the wheat leaves seen under visual phenotypic observation. 

The accumulation of certain cellular substances, known as stress markers, could be indicative for stress severity. Based on the elevated levels of proline and MDA in the leaves of UV-B-treated plants in our study, it could be concluded that einkorn experienced a higher degree of stress in response to the applied radiation. It should be noted, however, that 4PU-30 pretreatment had an alleviating effect on MDA and proline accumulation. Oxidative stress is considered a harmful aspect of UV-B impact primarily by triggering the formation of highly reactive species. Imbalances between ROS production and antioxidant scavenging capacity could provoke non-specific damage to DNA, proteins, and lipids [59]. The presence of elevated amounts of hydrogen peroxide is regarded as a symptom of impaired redox potential and increased oxidative strain [12]. On the other hand, ROS and membrane degradation products might play a role in mediating UV-B protection as well by modulating gene expression, proteolysis, and cellular redox dynamics [12,60,61]. Exogenously applied cytokinin 4PU-30 decreased hydrogen peroxide accumulation after UV-B treatment in wheat but failed to yield the same result in einkorn. Since einkorn maintained elevated hydrogen peroxide levels after 4PU-30 application and subsequent UV-B treatment, a limited effectiveness of 4PU-30 on reducing ROS formation could be assumed in this genotype. 

In general, abiotic stress increases the production of ROS, while CKs stimulate the antioxidant system to remove these toxic substances [17,60,61,62]. It is well documented that levels of endogenous CKs decline under stress and the exogenous application of CKs could improve plant performance through the amelioration of assimilate partitioning, retardation of senescence, and moderation of multiple metabolic processes [16,19,20]. Non-specific low-molecular-weight compounds like SH, phenols, and flavonoids might improve oxidative stress defense primarily by acting as free radical scavengers [55,63,64,65]. Polyphenols and free soluble phenolic acids increased in einkorn in response to radiation and were reported to inhibit lipid peroxidation [2]. In addition, UV-B exposure caused a fluctuation in the dynamic pool of redox interactions engaged in oxidative stress protection, which is maintained by multiple non-enzymatic antioxidants, including phenolics, carotenoids, cytochromes, ascorbate, glutathione, xanthophylls, α-tocopherol, polyamines, and proteins that carry redox-active SH groups [11,13,66]. In the present study, we detected an increase in total phenols in the leaves of wheat and einkorn plants subjected to excessive UV-B radiation and accumulation of thiols only in einkorn. Although flavonoid accumulation could serve as shield against UV damage, in our experiments, no significant increase in the levels of these substances was observed. Our results suggested that 4PU-30 increased free radicals’ scavenging ability (neutralization of DPPH•) under UV-B stress in both genotypes, which correlated with the accumulation of SH compounds in wheat and flavonoids in einkorn. Thus, exogenous 4PU-30 contributed to the release of strain experienced by the plants. Similarly, kinetin pretreatment was found to improve the cold stress performance of coffee plants through an increase in non-enzymatic antioxidants and enhanced radical scavenging capacity [48]. The plant antioxidant system is comprised of enzymes that either remove free radicals or prevent their formation. The first line of defense against ROS accumulation is superoxide dismutase (SOD), which converts the O^2−^ radicals to H_2_O_2_ [12]. We detected increased SOD activity in response to UV-B treatment in both studied genotypes, but this intensification was higher in einkorn compared to the respective control. Two other groups of enzymes, catalase (CAT) and peroxidase (POX), scavenge H_2_O_2_ but have different affinities to this toxic substrate. CAT has a low affinity whereas POX has a higher affinity to H_2_O_2_ and might act in the fine regulation of ROS levels [60,61]. Since Guaiacol peroxidase can act as an effective extinguisher for reactive oxygen intermediates and peroxide radicals under extreme conditions, different environmental stressors can induce its activity in plants [13]. Our results provided evidence that a major role of hydrogen peroxide degradation was played by POX since its activity was substantially increased due to UV-B radiation exposure. However, it is likely that different isoforms of these enzymes could contribute to the detoxification effect [35]. Glutathione S-transferase (GST) is also regarded as a “stress enzyme” due to its involvement in plant responses to drought, salt, and heavy metals [33,67,68]. It metabolizes toxic products of lipid peroxidation and enables the breakdown of xenobiotics (foreign compounds that are not naturally produced within the organism) in a coupling reaction with glutathione. We observed increased GST activity in both wheat and einkorn after exposure to UV-B. The higher SH-containing substances and MDA levels in einkorn corresponded to the more significant increase in GST activity in this genotype. 

Variations in the expression patterns of different antioxidant genes, as well as a differential transcriptional regulation in the two *Triticum* species, were observed. The most highly responsive genes found in our study were members of the *POX* and *GST* families. Interestingly, *POX1* and *POX7* have been implicated in both the biotic and abiotic stress response of einkorn plants, although their individual transcription pattern was stress-specific. Furthermore, only *POX1* was upregulated upon application of different signaling molecules playing important roles in regulating plant development and defense response [69]. In barley, *POX1* transcript levels showed a steady increase after UV-B treatment, while *GST* regulation differed depending on the growth stage of the seedlings [70]. In this work, *POX1* and *POX7* showed expression changes that differed significantly in UV-B- and 4PU-30-treated einkorn plants in comparison to the untreated controls. In wheat, *POX1* and *POX7* were also upregulated in seedlings exposed to combined UV-B and 4PU-30 treatment. These results were also in very good agreement with the increased POX activity observed at the protein level in UV-B-irradiated plants. A direct parallel between the two assays is not possible since the involvement of other *POX* isoforms cannot be excluded; however, such data imply a common response of the seedlings and suggest that the *POX1* and *POX7* genes may be required for the UV-B tolerance of the two species studied. 

Regarding the relative expression of the *GST* members assessed here, wheat and einkorn plants showed transcription patterns that were both species- and gene-dependent. The *GSTU6^1^* was significantly downregulated in all sample variants in respect to the control wheat seedlings. Similarly, the transcriptional profile of the *GSTL* gene was almost identical. On the other hand, *GSTU6^2^* was not affected by the UV-B treatment alone, although its activity increased upon irradiation when the wheat seedlings were pretreated with the plant regulator 4PU-30. Einkorn plants responded in a similar way in respect to the abundance of their *GSTU6^2^* related transcript. On the other hand, a completely different gene regulation was observed for *GSTU6^1^* and *GSTL* in the einkorn seedlings. The drastic increase in *GSTU6^1^* mRNA levels in both UV- and 4PU-30-treated samples implies that its respective gene product may play an important role in the recovery of einkorn plants from UV-B-induced oxidative damage. *GST* gene expression profiles did not correspond directly to the GST activity assessed here at the protein level, particularly in wheat. However, it has to be pointed out that the *GST* family is very large and diverse, with hundreds of *GST* genes identified in the genomes of *Triticum* species that vary in their gene functions and transcriptome characteristics depending on the growth, development, hormone regulation, as well as adaptation of plants to stress [67,68,71]. Thus, our data suggest an active contribution of certain *GSTU6* and *GSTL* genes in the activation of the UV-B-induced stress response and oxidative tolerance of plants studied here. Such a conclusion is consistent with the established ability of *GSTU7* overexpression to restore the oxidative stress response in UV-B-irradiated Arabidopsis plants [72]. 

In einkorn, the *TmCAT1* gene was implicated in the plant’s stress tolerance, with higher transcription in leaves, where an upregulation was observed three days after hormone application. However, in response to abiotic stresses, including H_2_O_2_ treatment, *TmCAT1* expression increased in roots but not in leaves [73]. Up to now, ten *CAT* genes have been identified and molecularly characterized in wheat, and a complex transcription pattern involving the up- and downregulation of different *CAT* genes under various conditions and stress treatments was also observed [74,75]. In our experiments, the *CAT1* gene showed quite similar expression profiles in both wheat and einkorn plants, where the transcript abundance decreased after UV-B treatment. Interestingly, a reduction in the CAT enzyme activity in the UV-B samples was also observed, particularly evident in einkorn, which is in full agreement with the UV-B response reported for field-grown wheat plants [76]. On the other hand, 4PU-30 application was associated with an increase in the enzyme activity in einkorn even in the UV-B-irradiated plants. Studies have shown that plant priming can boost *CAT* gene expression, otherwise inhibited by UV-B, and thereby stimulate the radiation response of soybean seedlings [77]. Taken together, our results indicate that as *CAT1* gene expression is negatively affected by both 4PU-30 and UV-B light, other *CAT* genes may contribute to the UV-B tolerance of einkorn and wheat seedlings under our experimental conditions. The relative stability of CAT enzyme activity in wheat samples may also be related to the potentially higher numbers of *CAT* genes existing in the hexaploid wheat in comparison to einkorn, thereby ensuring an increased stress resistance of this species. 

Three major groups of SODs are present in plants, classified based on their metal cofactor (MnSODs, FeSODs, and Cu/ZnSODs) and cellular localization [36,78]. Recent studies have discovered more than twenty *SOD* genes in the hexaploid wheat genome, categorized as three *MnSOD*s, six *FeSODs*, and the rest forming the largest group of *Cu/ZnSODs*, with constitutive and/or stress-modulated transcription patterns [38,79]. Therefore, in our work, we selected for expression analysis three *SOD* genes, each representative of a different *SOD* class. *SOD* genes showed diverse transcription profiles both in wheat and einkorn, dependent on the gene analyzed. In wheat, all three *SOD* genes were transcriptionally stable, even after UV-B radiation, with the exception of *MnSOD* only. The transcript levels of *MnSOD* increased in the 4PU-30-pretreated control and UV-B-irradiated samples. In einkorn seedlings, on the other hand, they were specifically modulated, i.e., *MnSOD* was upregulated and *FeSOD* and *Cu/ZnSOD* were downregulated in seedlings exposed to UV-B. While 4PU-30 application was associated with relatively stable transcript levels of *MnSOD* and *FeSOD*, the *Cu/ZnSOD* showed a reduction in these samples. At the protein level, SOD activity increased in both species irradiated with UV-B, with a more pronounced effect in einkorn. Thus, under our experimental conditions, SOD enzyme activity improved UV-B tolerance in both species, and a particular role could be attributed to the mitochondria-localized MnSOD. Notably, the *MnSOD* gene has been implicated in the oxidative stress response of *T. monococcum* seedlings [39] and the induction of specific MnSOD isoforms has been shown to confer protection against UV-B in soybean [77]. 

In conclusion, 4PU-30 pretreatment exerted a recuperating effect upon antioxidant enzymes triggered by UV-B stress as evidenced by the recovery of their activities to the control levels. Although, most probably, cytokinins alone could not provide adequate protection against UV damage, better performance of 4PU-30-pretreated wheat and einkorn plants under excessive irradiation was evidenced. Our results suggested that einkorn experienced a higher degree of stress in response to the applied UV-B radiation. These findings emphasize the differential responses of wheat and einkorn plants to excessive UV-B treatment, implying that the reaction towards this environmental stimulus could have a genotype-specific nature. 

Stress damage could be crucial especially during seed germination and at the early seedling stage. It was shown that 4PU-30 seed priming improved metabolic activity after cold storage, thus contributing to enhanced seed viability [80]. Exogenous CK improved growth performance by attenuating the damaging effects of UV-B radiation and stabilizing the photosynthetic apparatus [15]. The results presented here emphasize the positive effect of cytokinin pretreatment and offer convincing evidence that priming with 4PU-30 could successfully be applied for improving stress tolerance in cereals. Furthermore, fundamental research on plant coping mechanisms and the pursuit of possibilities for alleviating the negative effects of stress could have a substantial influence on future agricultural practices in view of the growing food demands of the human population. Although our work was focused on the harmful effects of UV-B, the latter was also reported to play a stimulating role on plant secondary metabolism [81], and the important regulatory functions of low UV-B levels have been recently reviewed for the purposes of plant cultivation [82]. Therefore, it should be specified that the impact of UV-B could be quite different depending on the dose and the duration of the irradiation. 

## 4. Materials and Methods

### 4.1. Plant Material, Growth Conditions, and Experimental Design

Wheat (*Triticum aestivum*, L.) from Bulgarian variety Enola and einkorn (*Triticum monococcum*, L.) seeds were grown for 10 days on half-strength Hoagland-Arnon nutrient solution under controlled conditions with a 25/21 °C day/night temperature, 16 h photoperiod, and 250 µmol m^−2^ s^−1^ photon flux density at leaf level. Afterwards, sets of one control and three test variants were established. Controls remained untreated while the following test groups were formed as follows: 4PU-30-treated plants, UV-B-treated plants, and plants subjected to a combination of 4PU-30 and subsequent 3 h UV-B illumination. Leaves of 10-day-old seedlings were sprayed with a water solution of 1 × 10^−6^ (1 µM) cytokinin 4PU-30 containing 0.1% Tween 80. Illumination with UV-B was imposed during the light period and 24 h after 4PU treatment. Seedlings were irradiated with UV-B lamps (Philips TL 2X20W/12 RSSLV/25, Amsterdam, Netherlands, λ_max_ 312 nm) for 180 min. The distance between the UV-B lamp and the top leaves of the treated plants was 0.12 m ± 0.04 m. Physiological and biometric parameters were measured 72 h after UV-B exposure. The first fully developed leaves were sampled for analyses and were either used immediately or frozen in liquid nitrogen and stored at −70 °C until measurement. 

### 4.2. Plant Biometry and Estimation of Leaf Pigment Content

The root length and length of the first fully developed leaf were measured from 10 plants of each variant. Results were given as means ± SD from two independent experiments (n = 20). For the estimation of pigment content in the leaves, fresh samples were homogenized in ice-cold 80% (*v*/*v*) acetone and were then centrifuged at 5000× *g* for 5 min at 4 °C. 

Chlorophyll (Chl a and Chl b) and total carotenoids (Car) absorption were measured spectrophotometrically at 663, 645, and 460 nm and pigment content was calculated by the equations of [83].

### 4.3. Remote Sensing Spectrometric Data

**Figure 8:**
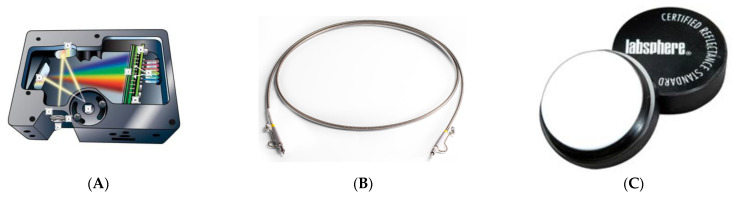
Hardware components included in the experimental setup: Ocean Optics (Dunedin, FL, USA) USB4000 spectrometer (**A**), durable fiber optic cable (**B**), and certified reflectance standard Labsphere (**C**).

The spectral reflectance characteristics of wheat and einkorn plants were measured using a remote sensing setup based on the following devices: Ocean Optics USB4000 spectrometer (Figure 8A) in the electromagnetic range 400 nm to 900 nm, Ocean Optics durable fiber optic cable (Figure 8B), and certified reflectance standard Labsphere (North Sutton, NH, USA) (Figure 8C). Remote sensing spectrometric measurements were performed with high spectral resolution (1–10 nm), allowing for the registration of reflectance spectra in the highest possible detail due to the better quality of the USB4000 sensor. All data were acquired by means of specialized software controlling the spectrometric device [84]. The illumination source used in the experiments was diffused sunlight and the integration time was adjusted in each experiment for the best dynamic range of the reflectance measurements. The durable fiber was always pointed at nadir to the measured sample.

### 4.4. Biochemical Analyses

Fresh leaf material collected from the first fully developed leaves was used for analysis or stored in liquid nitrogen until usage. Extraction was performed at 4 °C by homogenization of 300 mg samples in 0.1% (*w*/*v*) trichloroacetic acid followed by centrifugation at 15,000× *g* and the supernatant was collected for the quantification of oxidative stress markers and non-enzymatic antioxidants. 

#### 4.4.1. Oxidative Stress Markers Analysis (Free Proline, Hydrogen Peroxide, and Malondialdehyde) 

Free proline was derivatized using acid ninhydrin reagent for 60 min at 100 °C, absorbance was read at 520 nm, and the amounts were calculated from a previously plotted standard curve. Hydrogen peroxide (H_2_O_2_) content was evaluated spectrophotometrically based on its reaction with 1 M KI for 60 min in the dark as described in [85]. Absorbance was read at 390 nm and the amounts of H_2_O_2_ were calculated from a standard curve with known concentrations. Malondialdehyde (MDA) content was determined as thiobarbituric acid reagent product according to [86]. Absorbances were read at 532 and 600 nm and quantification was performed using a 155 mM^−1^ cm^−1^ extinction coefficient. 

#### 4.4.2. Non-Enzymatic Antioxidants (Total Phenolic Compounds, Flavonoids, and Free Thiol Groups)

Total phenolic compounds contained in leaf extracts were measured using Folin–Ciocalteu reagent supplemented with sodium carbonate, absorbance was read at 725 nm, and results were given as gallic acid equivalents (mg GA fresh weight g^−1^) according to [87].

Total flavonoid content was analyzed by the aluminum chloride colorimetric assay [64]. Absorbance was read at 510 nm and the results were expressed in rutin equivalents. Free thiol (SH) group-containing compounds were measured after the incubation of leaf extracts for 10 min at 20 °C in Ellman’s reagent according to [88]. Absorbance was measured at 412 nm. 

#### 4.4.3. Free Radical Scavenging Activity and Antioxidant Capacity

Free radical scavenging activity was analyzed in leaf extracts based on the antioxidants’ ability to bleach purple methanol solution containing DPPH• (1,1-diphenyl-2-picrylhydrazyl) that can readily undergo reduction by the antioxidants in the sample [89].

Radical scavenging activity (DPPH inhibition %) = [(A_control_ − A_sample_)/A_control_] × 100%,

where A_control_ is the absorbance of the control sample and A_sample_ is the absorbance of the test sample measured at 517 nm. 

Ferric reducing antioxidant power (FRAP) assays were carried out according to [90]. The method is based on the rapid reduction of ferrous-tripyridyltriazine (FeIII-TPTZ) to ferric-tripyridyltriazine (FeII-TPTZ) blue product at low pH by antioxidants present in the samples. Absorbance was measured at 593 nm and the results were given in μmol Fe^2+^ TPTZ g^−1^ FW, expressing ferric reducing activity equivalent to 1 mM FeSO_4_. 

A Multiskan Spectrum spectrophotometer equipped with a microplate reader (Thermo Electron Corporation, Vantaa, Finland) was used in these measurements. 

#### 4.4.4. Preparation of Crude Extracts and Assays for Antioxidant (ROS-Scavenging) Enzyme (CAT, SOD, POX, and GST) Activities

Antioxidant enzymes were extracted from leaf tissue. Fresh leaf material (500 mg) was homogenized at 4 °C in 5 mL of 50 mM potassium phosphate buffer (pH 7.8) supplemented with 10 mM ethylenediaminetetra-acetic acid (EDTA), 1 mM dithiotreitol, 0.1 mM phenylmethylsulfonyl fluoride (PMSF), and 1% polyvinylpyrrolidone. After centrifugation at 12,000× *g* for 30 min, supernatant aliquots were used for the assaying of specific enzyme activities [91]. Total soluble protein content was determined according to [92] using bovine serum albumin as a standard. 

Catalase (CAT, EC 1.11.1.6) activity was assayed as in [93] based on H_2_O_2_ decomposition measured spectrophotometrically by monitoring the absorbance decrease at 240 nm and using the molar extinction coefficient (39.4 mM^−1^ cm^−1^) in calculations. Results were presented as the amount of H_2_O_2_ decomposed per mg protein per min.

Total superoxide dismutase (SOD, EC 1.15.1.1) activity was estimated according to [94]. The method was based on the inhibition of the photochemical reduction of nitroblue tetrazolium (NBT). The reaction product was measured spectrophotometrically following the absorbance increase at 560 nm. SOD activity was defined as mg protein causing 50% inhibition of NBT photoreduction rate. 

Peroxidase (POX, EC 1.11.1.7) activity was assayed according to [95] using guaiacol as an electron donor. The formation of tetraguaiacol (TG) was monitored through the increase in absorbance at 470 nm and calculated using the molar extinction coefficient (26.6 mM^−1^ cm^−1^). Results were given in µmol TG mg^−1^ protein min^−1^.

Glutathione S-transferase (GST, E.C. 2.5.1.18) activity was determined by measuring the rate of conjugation of glutathione (GSH) with 1-chloro-2,4-dinitrobenzene (CDNB) according to [96]. The absorbance at 340 nm was monitored, a 9.6 nmol^−1^ cm^−1^ extinction coefficient was used for calculations, and the results were expressed as µmol reduced CDNB mg^−1^ protein min^−1^. 

Enzyme activities were measured on a Shimadzu UV-1601 spectrophotometer (Kyoto, Japan).

### 4.5. Gene Expression Analysis 

The experimental variants of einkorn and wheat seedlings subjected to gene expression analyses were as follows: controls (without any treatment and pretreated with 1 µM 4PU-30) and UV-irradiated (UV-B only and 1 µM 4PU-30 plus UV-B). The first leaf of 3–5 seedlings (equal to ~130 mg of tissue) was excised, wrapped in aluminum foil, immediately frozen in liquid nitrogen, and then stored at −70 °C for further processing. Frozen material was initially ground in liquid nitrogen and pure RNA was isolated with the RNeasy plant mini kit (Qiagen, Hilden, Germany) according to the manufacturer’s instructions with on-column DNase digestion. The RNA elution step was performed 3 times for each sample. RNA concentrations and purity were determined spectrophotometrically (BioSpec-nano, Shimadzu, Kyoto, Japan). Reverse transcription (RT) reactions for cDNA synthesis were performed with the FIREScript^®^ RT cDNA synthesis KIT (Solis BioDyne, Tartu, Estonia) according to the recommended protocol utilizing random hexamer primers and 1 μg of the RNA template. Each RT reaction was diluted 1:5 with ultrapure water and 2 μL of the diluted cDNA was used as a template for qPCR analysis. The final PCR reaction mixture (10 μL) was prepared with the HOT FIREPol^®^ EvaGreen^®^ qPCR Supermix according to the manufacturer’s protocol (Solis BioDyne, Tartu, Estonia). The RT-qPCR was carried out in a PikoReal Real-Time PCR System (Thermo Scientific, Waltham, MA, USA). The amplification conditions included initial denaturation for 15 min at 95 °C, 40 cycles of denaturation for 15 s at 95 °C, annealing for 30 s at 60 °C, and extension for 30 s at 72 °C, followed by final elongation for 5 min at 72 °C and melting curve analysis (60–95 °C, temp. increment 0.2 °C). All data were analyzed using the PikoReal Software version 2.2 (Thermo Scientific). The fold change in the expression levels of the genes was estimated based on Pfaffl’s method [97]. The *18S* ribosomal RNA fragment (118 bp) was used as a reference control amplified with the following primers: HV_18Ssh1F-CCTGCGGCTTAATTTGACTCA and HV_18SR- AACTAAGAACGGCCATGCAC [98]. The genes whose expression was characterized were *SOD*, *CAT*, *POX*, and *GST*. All of them are part of gene families; therefore, a selection was made based on the literature data for the involvement in stress response and/or the availability of DNA sequences for both species in the NCBI database. Primers utilized in the current study were either newly designed or taken from the literature. A detailed description of the primers, their target genes, and the respective references is provided in Table 3. Gene-specific primers were designed utilizing the Primer Blast Tool of the NCBI database (primer designing tool (nih.gov)) [99]. Primer pairs were designed to span exon-exon boundaries (where possible) to avoid the amplification of any residual gDNA contamination during qPCR. The newly constructed primers were aimed to target transcripts of the einkorn genome and all three wheat genomes (AABBDD) where possible. Primers were tested for specificity against the wheat mRNA Seq NCBI database and only those showing 100% specificity and no other potential targets in the wheat genome were selected. In addition, all primer pairs were tested for possible primer dimers by the online tool “Multiple Primer Analyzer” set to maximum or optimal sensitivity (Multiple Primer Analyzer/Thermo Fisher Scientific) [100]. 

### 4.6. Statistical Analysis

Two independent experiments were carried out and parameters were measured in three technical replicates. Data are given as means ± SD. The significance of differences between treatments was analyzed by one-way ANOVA with post hoc Duncan’s multiple range test at the 0.05 level. The statistical analysis of gene expression data was completed using Student’s *t*-test at the 0.05 probability level, based on the ΔCt values.

## 5. Conclusions

At an early developmental stage, wheat and einkorn seedlings responded to excessive UV-B radiation in a rather similar way by the activation of specific defense mechanisms. However, einkorn experienced a higher degree of stress revealed by intensified enzyme activity, increased accumulation of non-enzyme components of the antioxidant system, as well as the activated upregulation of gene expression. Although our preliminary hypothesis that einkorn would perform better under increased irradiation was not confirmed, it was demonstrated that synthetic cytokinin 4PU-30 pretreatment had the potential to alleviate the negative effects of oxidative stress by attenuating the symptoms of superfluous UV-B illumination in both studied genotypes. The use of remotely sensed spectral data represented a promising non-destructive technology for the detection of UV-B stress impact and plant recovery caused by 4PU-30, these findings being supported by several independent types of data registration.

## Figures and Tables

**Table 1:** Pigment content in the leaves of young wheat and einkorn plants illuminated with UV-B, sprayed with synthetic cytokinin 4PU-30, and a combination of the two treatments. Data are means ± SD (n = 10). Different letters represent statistical significance at *p* < 0.05.

Variants		Chlorophyll *a* (mg/g FW)	Chlorophyll *b* (mg/g FW)	Carotenoids (mg/g FW)
**Wheat**				
	Control	1.307 ± 0.036 *b*	0.452 ± 0.017 *a*	0.799 ± 0.026 *a*
	UV stress	0.845 ± 0.010 *d*	0.270 ± 0.003 *c*	0.510 ± 0.006 *c*
	4PU-30	1.419 ± 0.025 *a*	0.480 ± 0.011 *a*	0.848 ± 0.023 *a*
	4PU + UV	1.186 ± 0.015 *c*	0.394 ± 0.005 *b*	0.670 ± 0.010 *b*
**Einkorn**				
	Control	1.158 ± 0.038 *b*	0.392 ± 0.024 *b*	0.679 ± 0.024 *b*
	UV stress	0.756 ± 0.025 *c*	0.285 ± 0.011 *c*	0.559 ± 0.020 *c*
	4PU-30	1.281 ± 0.036 *a*	0.449 ± 0.015 *a*	0.750 ± 0.018 *a*
	4PU + UV	1.139 ± 0.036 *b*	0.395 ± 0.014 *b*	0.686 ± 0.023 *b*

**Table 2:** Gene expression data presented as a table. Control (no treatment), UV-B exposure (UV), application of 1 µM 4PU-30 (4PU-30), and combined treatment with UV-B and 1 µM 4PU-30 (4PU + UV). Results are presented as mean ± SE, n = 3. Significant differences compared to controls (*p* ≤ 0.05) are marked with asterisks (*) and highlighted in bold.

Genes	Treatments							
	Control		UV		4PU-30		4PU + UV	
	Wheat	Einkorn	Wheat	Einkorn	Wheat	Einkorn	Wheat	Einkorn
***MnSOD***	1	1	1.043 ± 0.090	1.894 * ± 0.030	1.365 * ± 0.120	0.95 ± 0.060	1.517 * ± 0.110	1.251 * ± 0.040
***FeSOD***	1	1	1.081 ± 0.050	0.616 * ± 0.030	1.326 ± 0.110	0.946 ± 0.020	1.206 ± 0.020	0.953 ± 0.060
***CuZnSOD***	1	1	0.906 ± 0.040	0.767 * ± 0.020	0.898 ± 0.060	0.65 * ± 0.060	1.081 ± 0.090	0.760 * ± 0.003
***CAT1***	1	1	0.667 * ± 0.050	0.641 * ± 0.020	0.763 * ± 0.030	0.531 * ± 0.040	0.946 ± 0.030	0.697 ± 0.050
***POX1***	1	1	4.301 * ± 0.680	11.457 * ± 1.050	4.038 * ± 0.510	3.762 * ± 0.150	5.155 * ± 0.820	10.147 * ± 1.630
***POX7***	1	1	1.615 ± 0.100	4.603 * ± 0.080	1.776 ± 0.130	0.691 * ± 0.030	2.564 * ± 0.060	2.588 * ± 0.130
***GSTU6^1^***	1	1	0.384 * ± 0.030	9.836 * ± 2.710	0.341 * ± 0.050	57.478 * ± 12.530	0.392 * ± 0.030	34.75 * ± 8.240
***GSTU6^2^***	1	1	0.939 ± 0.040	1.480 * ± 0.100	1.235 ± 0.020	1.212 ± 0.100	1.732 * ± 0.060	2.74 * ± 0.170
***GSTL10***	1	1	0.326 * ± 0.040	1.38 ± 0.030	0457 * ± 0.010	0.986 ± 0.004	0.547 * ± 0.020	1.366 ± 0.020

**Table 3:** Primer pairs and their targets in wheat and einkorn.

Primer Name	Primer Sequence	Reference	Amplicon Length	Target Gene Tm/Ta	Acc. No and Chromosome Localization (In Wheat)
MnSOD-F MnSOD-R	TCCGCCGTCGTCCACCTC CCACCACCCTCGCTGATG CCACCACCCTCGCTAATA (this study)	Karimi et al., 2017 [101]	104 bp	TmMnSOD TaMnSOD3.1	MK091461.1-Tm XM_044603645.1-Ta 2A
TaFe-SOD-F TaFe-SOD-R	CCTACTGGATGAGACGGAGAG GGACGAGGACAACGACGAA	Luo et al., 2019 [102]	124 bp	TaFeSOD	AK453889.1-Ta 7D JX398977.1-Ta 7D XM_044510132.1-Ta 4A
TaCu/Zn-SOD-F TaCu/Zn-SOD-R	TGGGAGAGCGTTTGTTGTTC GTCTTCCACCAGCATTTCCA	Luo et al., 2019 [102]	92 bp	TaCu/Zn-SOD, SOD1.2	XM_044573537.1-Ta 7A, X1 XM_044573538.1-Ta 7A, X2 XM_044573539.1-Ta 7A, X3 XM_044578041.1-Ta 7B, X1 XM_044578042.1-Ta 7B, X2 XM_044587054.1-Ta 7D
qTmCAT1-F qTmCAT1-R	CGAGAAGATGGTGATCGAGAA TGTTGATGAATCGCTCTTGC	Tounsi et al., 2019 [73]	95 bp	TmCAT1 TaCAT1	MK091459.1-Tm NM_001405704.1-Ta 4B XM_044520955.1-Ta 4D XM_044527958.1-Ta 5A
TGST2-F TGST2-R	TACGAGGACGTGGAGGAGAA TGTGGATGAGCACGGGTATC	This study	91 bp	TmGSTU TaGSTU61	Tm EF044232.1 XM_044552184.1-6A XM_044550330.1–6A
TaGSTU56-F TaGSTU56-R	TTAAAGATCTCGTCGTTCCAC AACAGCTACTCACAAGGCAGA	Wang et al., 2019 [71]	92 bp	TaGSTU62	XM_044570870.1-Ta 7A XM_044581608.1-Ta 7D
TaGSTL10-F TaGSTL10-R	ATGTGCCATTTATCGAAAGGT TCCATGCTGCAGTAGTTCCC	Wang et al., 2019 [71]	234 bp	TaIN2-1 homolog B	XM_044507158.1-Ta 4A XM_044514325.1-Ta 4B XM_044519678.1-Ta 4D, X1 XM_044519679.1-Ta 4D, X2
POX1u-F POX1u-R	CTCCAGGGTGAACTCGTGAT GCCTTTGCATGAGAAAGTGGG	This study	219 bp	TmPOX1 TaPOX1	AY857755.1-Tm XM_044599368.1-Ta 2A XM_044466236.1-Ta 2B XM_044474478.1-Ta 2D
POX7u1-F POX7u1-R	GTCGTGGACGAGGTCAAGAG TGGGTCCACCAGTCAGCA	This study	112 bp	TmPOX7 TaPOX17-like	AY857761.1-Tm XM_044525767.1-Ta 5A XM_044533224.1-Ta 5B XM_044541856.1-Ta 5D

## Data Availability

Data in this research are contained within the manuscript tables and figures.
